# 3D Virtual Reconstruction of the Ancient Roman *Incile* of the Fucino Lake

**DOI:** 10.3390/s19163505

**Published:** 2019-08-10

**Authors:** Luca Di Angelo, Paolo Di Stefano, Emanuele Guardiani, Anna Eva Morabito, Caterina Pane

**Affiliations:** 1 Department of Industrial and Information Engineering and of Economics, University of L’Aquila, via G. 6 Gronchi 18, 67100 L’Aquila, Italy; 2 Department of Engineering for Innovation, University of Salento, Lecce 73100, Italy

**Keywords:** remote sensing, monumental heritage, archaeology, 3D virtual reconstruction

## Abstract

The construction of the artificial emissary of Fucino Lake is one of the most ambitious engineering buildings of antiquity. It was the longest tunnel ever made until the 19th century and, due to the depth of the adduction inlet, it required a monumental and complex *incile*, which, for functionality, cannot be compared to other ancient emissaries. The Roman emissary and its "*incile*" (Latin name of the inlet structure) were almost completely destroyed in the 19th century, when Fucino Lake was finally dried. Today, only few auxiliary structures such as wells, tunnels, and winzes remain of this ancient work. As evidence of the ancient *incile* remains a description made by those who also destroyed it and some drawings made by travelers who, on various occasions, visited the site. This paper presents a virtual reconstruction of the Roman *incile*, obtained both through the philological study of the known documentation, interpreting iconographic sources that represent the last evidence of this structure, and through the survey on the territory. The main purpose is to understand its technical functionalities, the original structures, and its evolution during the time, taking into account the evolution of the Fucino Lake water levels, technological issues, and finally offering its visual reconstruction.

## 1. Introduction

**Figure 1:**
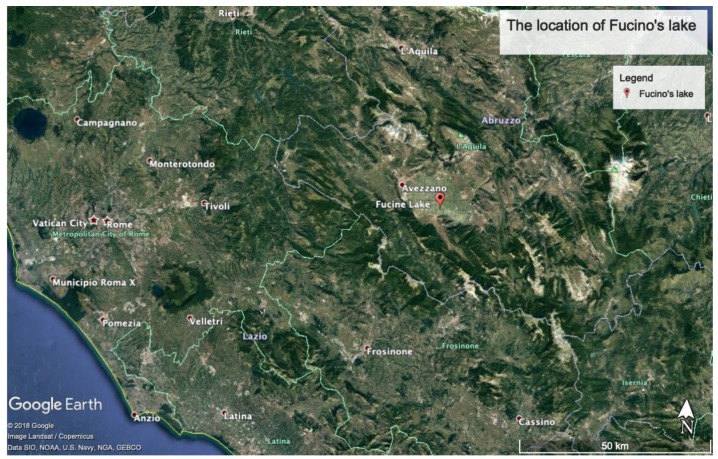
The location of Fucino Lake.

The Latin word *incile* is used to identify an opening or channel for the passage of water, in order to regulate or to fully drain a water basin, e.g., a lake. The word probably derives from the Latin verb “*incidere*”, which means to cut, to move, or to put in motion. From a functional point of view, an *incile* is an accessory of a drainage system, which serves to retain waters during the construction of an artificial emissary or, afterwards, to control the water drainage when the emissary is fully functioning. Fucino’s *incile* was a complex structure with different sub-systems situated on Fucino Lake, about 100 km east of Rome (Figure 1). This construction evolved over time (from its initial construction to the final lake desiccation) by changing its configuration, as well introducing new parts once the water was drained and the level of the lake was decreasing. In other cases, the *incile* was a relatively simple structure, such as the one of the Lake Trasimeno (from Etruscan/Roman period) where the artificial emissary is an open channel and not an underground tunnel as in the case of the Fucino's emissary.

Although the ancient Romans and Etrurians built other emissaries as underground tunnels, such as those for the lakes of Nemi (late 6th/early 5th A.C. century) and Albano (395 A.C. circa), Fucino’s emissary was more difficult to realize since there was more water to emit and the emissary was much deeper (the underground tunnel was 15 m under the water level). 

Historic sources on ancient *incilia* are generally very few, even on the important ones, such as Nemi and Albano. Recent studies, such as those conducted by the Commission on Artificial Cavities of the Italian Speleological Society [1], tried to map, register, and describe the main features of the ancient underground hydraulic works throughout the Italian territory. Despite the presence of various emissaries, not one has an *incile* that can be compared to the Fucino one.

Fucino Lake’s emissary and its *incile* were realized by Romans between 41 and 52 B.C. The main reason was in the Fucino endorheic nature, which means that it had no natural emissary. For this reason, the water level had great variations, overflowing onto the surrounding lands and causing several problems for the population living near its shores. The aim of the ancient emissary of Fucino was to reduce the lake’s extension, control, and regulate its water level. 

The Fucino emissary built by Romans had a length of about 5650 m and it was replaced only in the 19th century, when the Roman emissary was entirely obliterated by the construction of a modern tunnel. The new emissary was made following the track of the old one, replaced by sections with a different shape, more suitable to withstand soil pressures and capable of a greater water flow. It is possible to see traces of the old emissary just in the section beneath Mount Salviano, under which the tunnel is located. The old construction, excavated by hand, intersects the new one excavated with mines. The only buildings that remained untouched are the Roman passages and descents that were also used to carry out the 19th century works [2].

The modern works, besides destroying the Roman *incile*’s structures, changed its position and orientation. In fact, Torlonia’s *incile* is placed further away compared to the ancient building, probably reusing some of the walls that composed it. Therefore, the Roman *incile* was replaced in its functions by a modern one, more monumental, and best suited to manage the massive water flow towards the larger new emissary.

The ancient roman *incile* had a complexity in its planning that cannot be found in other ancient structures performing the same function. Currently, nothing remains of this work, and nothing is known about the phases of construction and maintenance that affected its evolution during the time. At present, there is only a description of the *incile* [3]*,* performed with orthogonal projections by the engineers that destroyed it in the 19th century. This description is partially incomplete and must be integrated with available iconographic documentation. To fully understand the *incile* functionality, however, a relocation to the environment within which it was used is required, also taking into account the water levels that changed during the time. In this paper, an original 3D virtual reconstruction of the ancient *incile* of Fucino Lake is presented. This operation required the integration of iconographic sources and land surveys performed by traditional technology based on measurements carried out by a theodolite, supplemented with land photogrammetry carried out by drone.

## 2. Related Works

In recent years, advances in data capture techniques and reality-based representations, together with the availability of low-cost virtual reality (VR) systems, have led to an in-depth review of the documentation methodologies for cultural heritage (CH). Additionally, 3D documentation techniques are taking over traditional 2D documentation, while data acquisition and processing are now widely implemented within IT (information technology) platforms, based on geographical information systems (GIS) or building information modeling (BIM) technologies, depending on the CH scale [4]. The 3D digital mock-up of historical monuments, buildings, and archaeological remains is integrated nowadays with new solutions for the representation and visualization of data relating to the urban and landscape environments with considerable repercussions in CH management, analysis, reconstruction, and restoration. Several geo-technologies, such as aerial and terrestrial photogrammetry, airborne and terrestrial laser scanning, mobile LiDAR systems (MLS) and global navigation satellite systems (GNSS) can be used in order to capture the shape, dimensions, and visual appearance of CH objects and sites at their current state. The growing integration between these technologies has allowed developing a multi-resolution approach in CH documentation [5]. This approach aims to return, through data characterized by a variable resolution, metric documentation consistent with the different geometries that have to be represented. CH objects, in fact, can range from details of small artefacts, from decorative motifs [6] to sculptures or buildings, and frequently, on the same site, structures with complex and rich detailed surfaces occur together with simpler shapes. The suitability of the various metric data sources must be evaluated based on several factors, among which are the accuracy required, the measuring instrument's portability, the size of the CH object and its complexity [7] so that there are no standard measurement technologies and methodologies valid for every possible situation. The use of integrated approaches and technologies represents the optimal solution especially for large and complex CH sites [8], where the peculiarities of the various techniques are combined and balanced together to reach an efficient solution within typical multiscale heritage contexts [9]. 

Nowadays, two main 3D data acquisition methods are used to generate 3D reality-based models from CH objects and sites: Range-based and image-based techniques, which use range sensors and imaging, respectively. Range sensors [10], such as triangulation-based, TOF (time of flight) or phase-shift scanners, directly capture the 3D geometry of an object, providing high-density 3D point clouds as output. Due to the object size, shape, and obstacles of the surrounding environment, more scans are necessary to cover the entire surface of an object or architectural structure. Since the 3D data have to be registered in a single coordinate system, the alignment and integration of different scans represent an important step of data processing, which can affect the final accuracy of the 3D model. Several registration techniques are available, most of which are based on the iterative closest point (ICP) approach. Other steps of data processing include noise reduction, outliers’ removal, and holes filling. Once the 3D point cloud has been cleaned and structured, a consistent tessellated surface is created to build a realistic representation of the modeled scene. A photorealistic visualization can afterwards be generated by texturing the virtual model with image information [11]. 

The 3D data capture methodologies based on imaging sensors, such as photogrammetry, and are known in the literature as image-based modeling techniques. Since the images are required before 3D measurements can be performed, these are indirect data acquisition methods. These methods provide realistic looking models and are generally preferred in the cases of objects, monuments, or architectures with regular geometric shapes, low budgets, good experience of the working team, time, or location constraints for the data acquisition and processing [12]. Since imaging sensors generate image data as output, a very important step of data processing, called image-matching, is required to retrieve the 3D object geometry in the form of a high-density 3D point cloud. Using the mathematical model of collinearity, photogrammetry establishes a geometric relationship between images (image space) and the real scene at the time of photographic shooting (object space). Once this relationship is reconstructed, it is possible to obtain metric information on the size, shape, and position of an object or scene starting from different homologous points identified in at least two images. Recent developments due to advances in computer vision have introduced a high level of automation modifying the photogrammetry approach with respect to the past [13,14]. However, georeferencing and scaling of the models still remain a manual task. Nowadays, image-based methods implement several types of image-matching algorithms [15], in order to establish the correspondences between two or more images. This phase of data processing is very important because it provides a depth map in the image space, whose corresponding result is the 3D point cloud in the object space. 3D measurement from images obviously requires that points of interest or edges are visible in the images. This is often not possible due to obstacles or self-occlusions or because there are no signs, edges, or features to be extracted. The final accuracy of the 3D model is also affected by lighting or by ambient light problems. 

Range and image-data are often combined together in order to generate a 3D multi-resolution representation able to derive different geometric levels of detail (LOD) of the scene under investigation. Besides range and image-data, topographic surveys of the area with differential GPS and archival maps are usually combined for placing all the 3D acquired data in the same georeferenced system [16]. Additionally, a complete description of the CH object often requires the reconstruction not only of its 3D geometry but also of its original location relative to the surrounding context, depending on the documentation and conservation purposes of the specific survey [17]. In that respect, particularly interesting are the UAVs (unmanned aerial vehicle), i.e., model helicopters which fly in an autonomous mode, using amateur digital cameras and GPS systems, and which can be used to get images from otherwise hardly accessible areas. 

In recent years, 3D digital documentation has assumed an additional interesting role in the CH domain for the possibility of visualizing historical monuments and architectures no longer existing. This is generally achieved through an analytical process, referred to as virtual anastylosis, based on the integrated knowledge of historical and 3D metric data [2]. The virtual anastylosis, in fact, aims at generating the 3D reconstruction of ancient architectures, nowadays partially or totally destroyed, starting from the 3D multi-resolution representation of the site at its current state and from historical and archival sources. These sources very often have non-metric properties and are of heterogeneous type since they appear, in analogue or digital format, as maps, texts, paintings, drawings, engravings, and old photographs. The starting point of the method is the 3D reality-based model that, by means of in-depth philological research, is used for generating the first reconstructive step, which usually represents a rough approximation of the architectural structure. Thanks to the interaction between archaeologists and CAD operators, archaeological considerations can be compared with geometrical constraints, producing a reduction of the reconstructive hypotheses to a limited set, each one to be archaeologically evaluated. This iterative refinement on the reconstructive choices is repeated until the result becomes convincing by both points of view, integrating in the best way all the available sources. The application of this approach allows for a better-shared solution between 3D metric data, historical sources, and archaeological knowledge [18]. This method has been applied in the virtual reconstruction of different CH contexts, such as, to cite only a few examples, a vast archaeological area located in central Vietnam [19], the late Roman Circus of Milan [12], or a tower of historical interest reconstructed by photogrammetry using only historical images [20].

The 3D virtual reconstruction of non-existent structures is particularly suitable with AR systems. AR technology enhances the perception of the real world by overlaying digital contents on the real world, with the aim of persuading the viewer that the virtual object is part of the real environment [21]. Archaeological sites can certainly benefit from this kind of application. By combining historical and archaeological details, for example, the user experience can be improved by imaging the archaeological ruins with the ancient landscape [22]. The diffusion of software platforms for the development of the AR experience, made the use of mobile augmented reality (MAR) devices also possible in the field of historical architecture [23]. The virtual anastylosis of complex architectural structures can be visualized nowadays as superimposed with archaeological remains of the structure. This 3D virtual reconstruction, in the form of a high-density polygonized model with texture, is often too heavy for allowing the 3D real-time navigation into a mobile augmented reality (MAR) platform. Now, a critical key point is to simplify the 3D model, maintaining its details and quality when it is exported in a mobile environment [24].

## 3. The Iconographic Sources

The lack of complete and reliable information concerning the ancient *incile*, together with its complete destruction, is not an obstacle of a philological method providing a valid reconstruction of this hydraulic construction. Thanks to Torlonia’s engineers’ description of the *incile* [3], integrated with other sources and historical data about lake waters levels, it is possible to attempt a reconstruction of how the *incile* looked, the way it worked, and to analyze the functions of its different components. The virtual reconstruction of this hydraulic work, in addition to visually return a long-lost technological and engineering heritage, allows contextualizing Claudio’s original project in the real territory. This permits the consideration of the operative conditions as well, in order to understand the early extension of the first drainage. 

**Figure 2:**
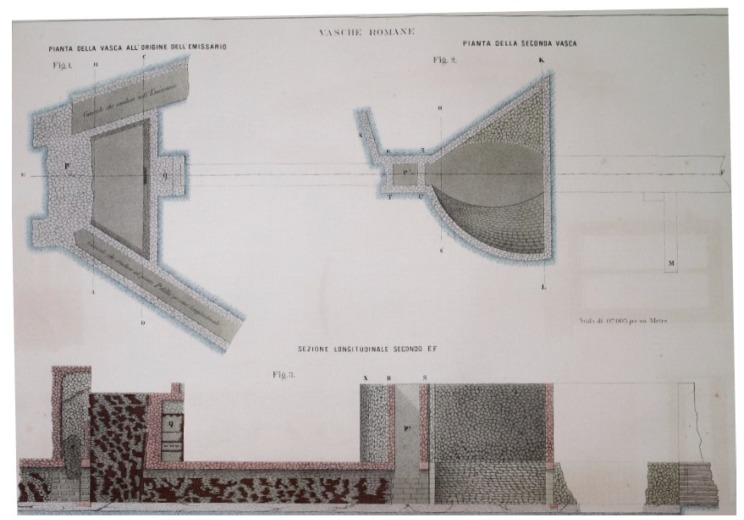
Plan and longitudinal section of the Roman *incile* by A. Brisse and L. De Rotrou [3].

Ancient literary sources refer to the *incile* mainly in relation to the inauguration ceremony of the Claudian emissary. There are few or no technical details, concerning both construction phases and the leveling, that can contribute to its reconstruction. The ancient *incile* was entirely destroyed with the 19th century works of the new tunnel built by the engineers who, on behalf of Prince Torlonia, took care of the construction. The ancient outlet has been replaced by a new monumental *incile*, built in a different position from the ancient one. Before proceeding to its destruction Brisse A. and De Rotrou L. documented the Roman *incile* with engineering drawings, plans, and sections. Therefore, the most complete information about the Roman *incile* is provided by the iconographic testimony left by Torlonia’s works executers, together with their written commentary, which describes plans and sections produced by them. The published Atlas Torlonia [3] collects all the testimonies concerning Claudio's emissary, allowing for a deeper knowledge of ancient *incile* and its subsidiary structures. As regards to the *incile*, it is possible to find a plan (Figure 2) and a longitudinal section plus four transversal sections, which illustrate the access point of the tunnel and the maneuvering chambers. 

**Figure 3:**
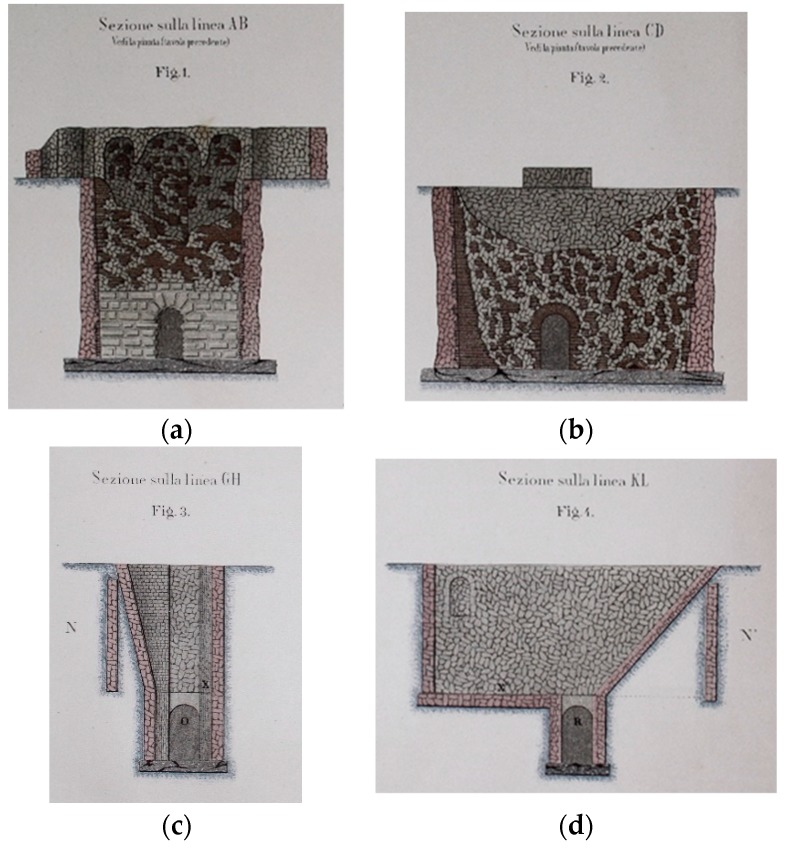
Sections of the Roman *incile*, by A. Brisse and L. De Rotrou [3]: (**a**) section AB of Figure 2; (**b**) section CD of Figure 2; (**c**) section GH of Figure 2; (**d**) section KL of Figure 2.

The drawings come with a graphical representation of the construction material that composed the *incile* (Figure 2 and Figure 3).

Although these drawings are also supported by a commentary, some incompleteness can be evidenced especially as regards to the two access ramps to the tunnel and to the gate control chamber. In addition to this iconographic production, specifically representative of the Roman work, a plan and a section of the first phase of the Torlonia’s works can also be found, in which the Roman *incile* is still partially maintained and integrated with modern constructions, before being definitely destroyed (later described in the Figure 8 of this work). This information has been used to refer the location of the ancient *incile* with the existing structures of the emissary.

The description by Atlas Torlonia, incomplete but of fundamental importance for the reconstruction of the *incile*, needs to be integrated with other iconographic material. Luckily enough, the Atlas of Torlonias’s emissary construction is not the only graphic record: Other iconographic sources describe the structure of the *incile*, with different levels of details.

**Figure 4:**
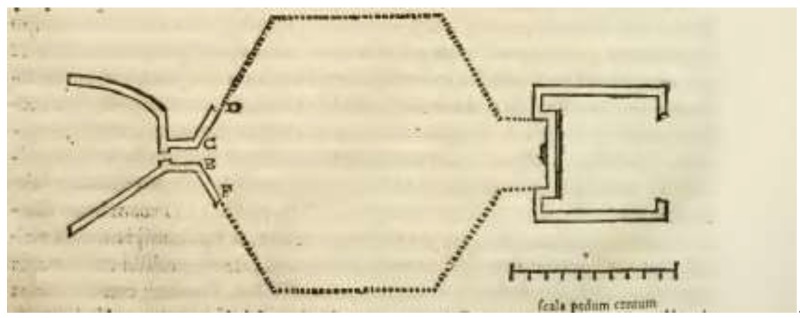
Plan of the Roman *incile* realized by R. Fabretti in Descrizione dell’emissario del Fucino 1683 [25].

Chronologically, the first known iconographic representation of the *incile* is R. Fabretti’s one (1682) [25]; he was able to witness firsthand the remains of the Roman hydraulic tanks (Figure 4). However, most of the drawings represent his personal hypotheses, because the *incile* was partially covered in soil at that time.

**Figure 5:**
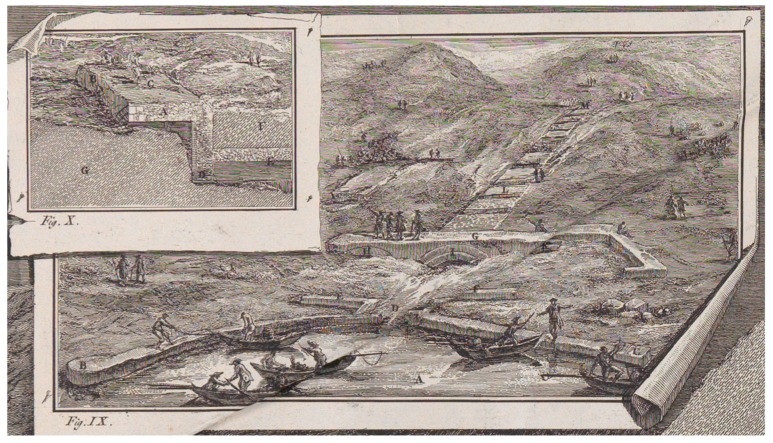
Representation of the Roman *incile* executed by F. Piranesi in 1790.

The work of F. Piranesi (Figure 5) represents a fundamental source, one of the most detailed, comprising several frames, which describe both the emissary and the *incile*. The remains of the latter are drawn reproducing what was seen in 1790 and integrating it with interpretations of what could not be seen. At that time, the *incile* was covered by soil sediments, but some parts were visible (Figure 5). The most interesting detail reveals a path behind the *incile’s* structures, probably connected with a later cancelled entrance. This gateway is evidenced in Atlas Torlonia drawing’s (Figure 3a). Furthermore, it is possible to observe that the three arches were covered at the intrados with double-row bricks, a detail that is not reproduced in 19th century drawings (Figure 3a), perhaps because at that time they had been removed and did not exist anymore.

**Figure 6:**
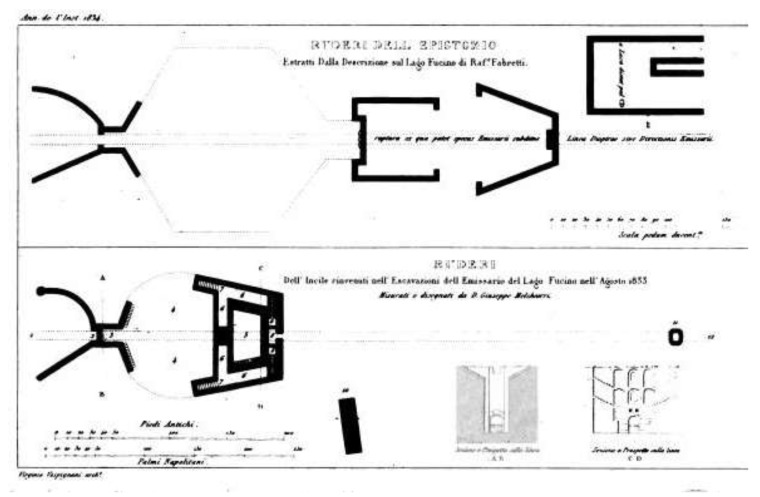
Annali dell’Istituto di Corrispondenza 1834, G. Melchiorri plan and front of the Roman *incile.*

In an issue of the Annali dell’Istituto di Corrispondenza (1834) it is reported a plan of the Roman *incile* realized by G. Melchiorri (Figure 6). It includes all of the *incile*’s structures, with a representation of the back wall of the trapezoidal tank: Here the author assumed two rows of arches, together with staircases connecting them.

**Figure 7:**
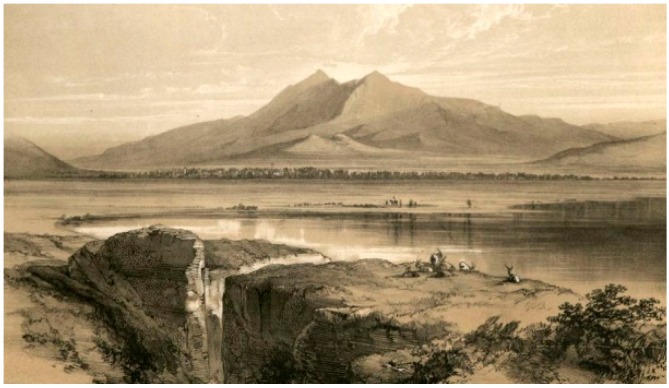
E. Lear 1843, Fucino Lake view with *incile* detail [26].

The iconographic sources include an artistic representation of Lake Fucino, with a perspective from the *incile*, realized by E. Lear (Figure 7). This sketch is very useful for the reconstruction, because it provides some information about building materials, structures, and the status in which the *incile* was before being destroyed by the 19th century works. Another iconographic source is a sketch performed by R. Caracciolo. It is a rough representation of the *incile* with some shoring, probably done while some works for removing the soil covering were being executed, at the beginning of the 19th century.

## 4. The 3D Virtual Reconstruction

### 4.1. Quantitative Description

Lake waters were conveyed into a collector channel, which ended at the *incile* where the waters were introduced into the emissary. On the lakeside of the *incile* there was the elliptic tank, the first tank of the outlet. In the deepest part, this corresponded to an elliptic basin, which, in the upper part, widened into a triangular-shaped volume. The south wall was curved towards the inside of the basin, giving the tank a peculiar shape (Figure 2). The structure had, in the center of the bottom part, an opening with a second gate adjustable by a maneuver room placed immediately above the collector tunnel. This tunnel ran below all the *incile*’s structures to the last tank: the trapezoidal tank. The water coming from the lake, after passing the second gate, fell into a third tank, smaller than the previous ones, but deeper: the trapezoidal tank, from the actual geometric shape it has in Brisse and De Rotrou’s plan and in all the other representations. On the longest side of the trapezium, there was a gate used to regulate or to block the water flow coming from the lake before it went inside the tunnel of the emissary. 

Just after the elliptical tank, a short passage (narrow 3 m, long 8 m, and deep 10.5 m) connected the first tank with a presumed large basin. The shape of the latter is not exactly known, since Brisse and De Rotrou did not consider it necessary to excavate its entire perimeter. The other iconographic sources, such as Fabretti, Piranesi, and Melchiorri, who describe this intermediary basin assuming that it had a hexagonal configuration, could only speculate about its shape since it was probably covered with soil. 

As regards to the functionalities of the *incile's* tanks, they probably had to serve as the basin of expansion and, slowing down the running water, for decanting, collecting, and removing the various debris that had managed to pass there. The same could be for the presumed hexagonal tank.

The *incile* illustrated in Torlonia’s Atlas, also described in other iconographic sources, was the *incile* in its final configuration. Most likely, this work has changed over time during the drying phases of the lake and the tanks were built in different phases, adapting the *incile*’s structure to the level of the lake’s waters gradually receding. The final configuration may have taken decades to be achieved. The alleged hexagonal tank may have existed in a specific phase of the Roman drainage work and, when no longer necessary, it was destroyed and then other parts of the *incile* were built.

In the virtual reconstruction of the *incile* some hypotheses have been made, especially about the area behind the larger side of the trapezium constituting the tank. Its internal articulation is, in fact, only partially illustrated in Torlonia’s Atlas and some fundamental data necessary for its reconstruction are missing. The first problem, regarding this tank, is the access to the maneuver chamber. According to the description expressed by Brisse and De Rotrou, there were two different tunnels. The north one drove directly to the artificial emissary, while the south tunnel led to the area with the emissary’s gate valve. However, Torlonia’s engineers did not care about fully describing these areas, so it is not clear how they were originally built. In the first edition of the Atlas, written in French and English, Brisse and De Rotrou describe the north entrance as a descending tunnel, while the south one is addressed as a short tunnel, with a staircase directly connected to the floodgates room. The filling and sanding of a large part of the *incile*, especially the central basin, make the reconstruction more difficult, multiplying the possible hypotheses.

The back wall of the trapezoidal basin, leant against the slope of Mount Salviano, was the head of the emissary tunnel, where two distinct parts were distinguished. At the bottom, there was the entrance to the emissary tunnel, whose floor was at the same level as the bottom of the trapezoidal tank. A third gate, adjustable by an overhead operating room, possibly served the entrance to the tunnel. A special service staircase probably connected the tunnel for possible inspections and maintenance interventions, once the water flow was blocked by means of the third gate.

In the upper part there were service rooms spread over two floors: In the lower floor, there was the maneuvering room for the third gate. The two floors were presented with a three-arched loggia-like structure. In the virtual reconstruction proposed here, we assumed that the three arches were built with two rows of bricks, using the typical measures of the Roman bricks for them. 

On the back of this upper wall, behind the three arches, it is possible to see in the detailed Torlonia section, a door or an old passage bricked up (Figure 3a). There is no clear evidence that can define and explain its possible role. Nevertheless, in Piranesi’s print (Figure 5) a pathway is represented with several steps. It can be assumed there was an access route with a ladder walled opening.

The philological reconstruction of this now lost structure would be very useful also for studying the *incile* functionality and understanding the most innovative knowledge of Romans, at that time, about the technologies used for lake drainage and reclamation works. For this purpose, the data concerning the lake waters levels are fundamental. Some information about the levels and depth variations of the Fucino waters (performed during the period in which regular measurements were made), comes from the middle of the 18th century, up to the final draining of Fucino Lake in the 19th century. There are also studies that speculate on the variations of the waters of the lake even in previous centuries [27]. The variations of the water levels, from 1783 to 1875, are shown in the Brisse and De Rotrou Atlas. 

### 4.2. The Relocation of the Roman Incile 

**Figure 8:**
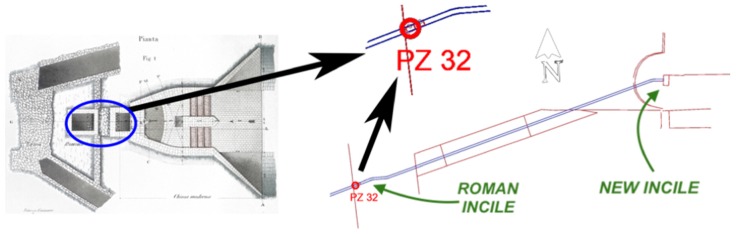
Analogies between Torlonia's modified *incile* and Torlonia’s emissary.

**Figure 9:**
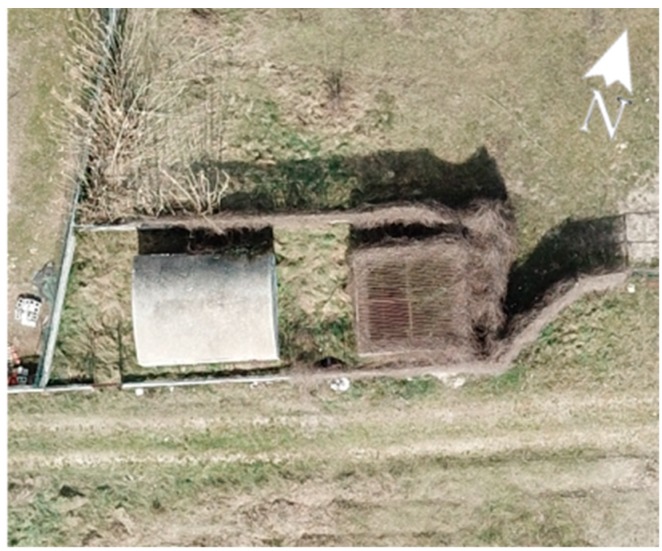
The external parts of the well n°32 built in the 19th century.

Today there are no ruins that can provide evidence as to where exactly the ancient *incile* was located. Furthermore, the area has changed a lot during the last centuries and deep alterations occurred in the territory, especially due to the 19th century works and modern human activities, such as agriculture or urbanization. Analyzing the historical sources and the current structures, a hypothesis has been formulated here. Torlonia’s drainage works envisaged, in an intermediate phase, the reuse of the *incile* with some modifications of the ancient structures. Some parts of Torlonia’s modified *incile* correspond to the well n°32 of Torlonia’s final emissary. In Figure 8, the ancient emissary’s drawing is found, modified by the addition of two wells (evidenced with a blue elliptic) and the new structures are shown on the right, according to Brisse and De Rotrou’s representation. Today the well n°32 is still present in the territory and can be observed in the countryside near the place where the *incile* was situated (Borgo Incile di Avezzano, AQ) (Figure 9). In order to place the *incile* in the territory, an emissary’s survey has been performed with traditional technology (Figure 8), while the surrounding area was detected with UAV photogrammetry.

Since the *incile* is part of a significant hydraulic work, the quality of geographical data is very important especially with regards to the elevations. For this reason, accurate data are necessary, particularly considering that the aim of the *incile*’s reconstruction is to investigate the hydraulic opera’s functionalities. Consequently, the territory data, provided by traditional surveys, are integrated by aero photogrammetric data.

The territory has been acquired by DJI Mavic Pro camera, whose technical specifications are reported in Table 1. 

**Figure 10:**
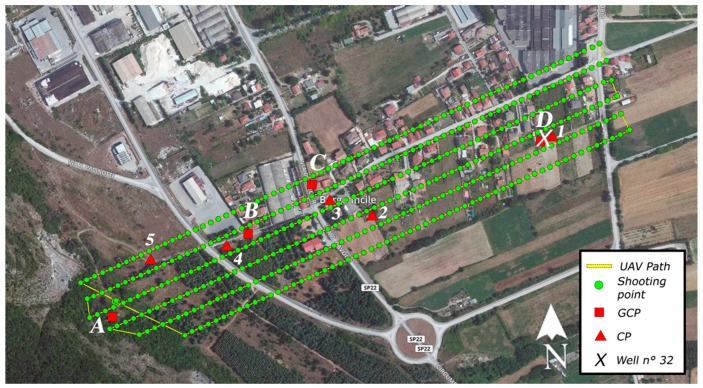
Flight plan used for photographic acquisitions in Borgo Incile di Avezzano (AQ).

The UAV is equipped with a GNSS sensor (GPS + GLONASS), whose accuracy is equal to a few meters. The entire area, which starts from the well n°32 to the Cunicolo Maggiore, was detected (Figure 10). This is an area 300 m large and 1000 m long. The flight altitude was 70 m with respect to the ground of the starting point (Cunicolo Maggiore). A lower value could not be used due to the presence of orographic obstacles near the Cunicolo Maggiore. Anyway, a ground simple distance (GSD) of about 4 cm/pixel was obtained for a large part of the map, which includes the area of major interest around the well n°32. The obtained accuracy of cloud point is generally greater than the GSD value and this value of repeatability can be considered adequate for the application in this work. Nevertheless, to improve the quality of the model, 5 ground control points (GCPs) were used as reference and two of them, in particular, were assigned to external solid elements of the emissary. Those points, distributed over the whole area of interest, allow to georeference the model and permit to scale exactly it. They were chosen between the fiducial points provided by the territory’s Agency of L’Aquila province (https://www1.agenziaentrate.gov.it/servizi/Monografie/ricerca.php). A key-feature of those points is that they are clearly distinguishable and consequently the use of markers for detecting them from the photos was not required. Six control points (CPs), moreover, were identified, whose altitude is known. Some of them were marked with the use of 30 × 30 cm squares.

All the previous information was taken into account for defining the flight plan, which has been created using the web-based application DroneDeploy. The map of the flight plan is evidenced in Figure 10. In that image, the yellow line represents the drone’s path, while the green points identify where photos have been captured. These photos were taken with an overlap of 75% for the front and of 65% for the side. In order to minimize the distortion effect, the viewing angle θ of the camera was fixed to 0 deg for all the photo acquisitions. Consequently, some details of the vertical walls of the surrounding houses were not captured during the survey, but they were considered as irrelevant for our final goal. Table 2 reports a summary of the parameters of the UAV survey. In Figure 10, the square points correspond to the GCPs and the triangular points identify the CPs.

At the end of the mission, about 390 photos were acquired, which have been processed in the software Pix4D (developed by Pix4D SA, Route de Renens 24, 1008 Prilly, Switzerland), one of the most used photogrammetry software. During the initial processing, all the information concerning the camera and the photos were extracted, such as orientation of the camera, focal length, geolocation info, etc. Then tie points were identified by the use of a bundle adjustment approach. At the end a densified point cloud was obtained, which was used to create the 3D textured mesh. The settings parameters are quoted in Table 3. 

**Figure 11:**
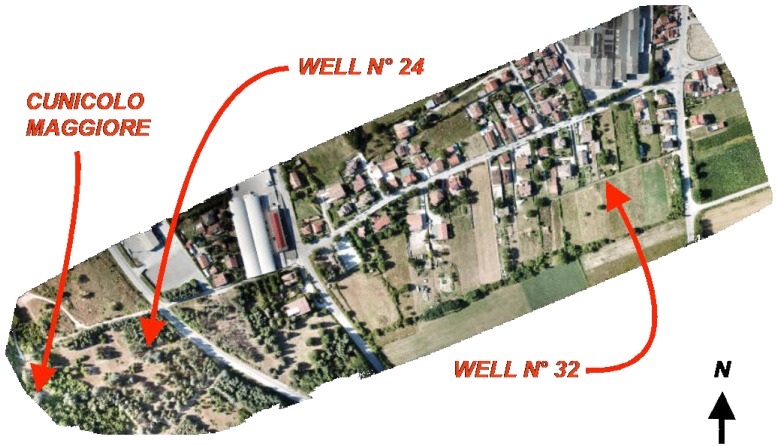
Point cloud resulting from photogrammetry elaboration.

After about 4 h of elaboration by using an Intel Xeon CPU E5-2680 (64 Gb RAM) work-station, the point cloud was obtained (Figure 11). 

The scaling and rototranslation of the point cloud were carried out by using as reference three points, two of them located respectively on the well n°32 (point D of Figure 10) and the Cunicolo Maggiore (point A of Figure 10). The other point (point C of Figure 10) is located outside the tunnel alignment. The well n°32 and the Cunicolo Maggiore are reference elements of the Emissary and they were used by ancient Romans to determine the direction and the depth of the Emissary tunnel. It was for this reason that the point cloud was transformed using these elements instead of other geo-referenced points. The transformed point cloud was verified by checking the elevations of the 5 CPs which are referenced in altitude. In Table 4, the errors for elevation (in meters) are reported.

**Figure 12:**
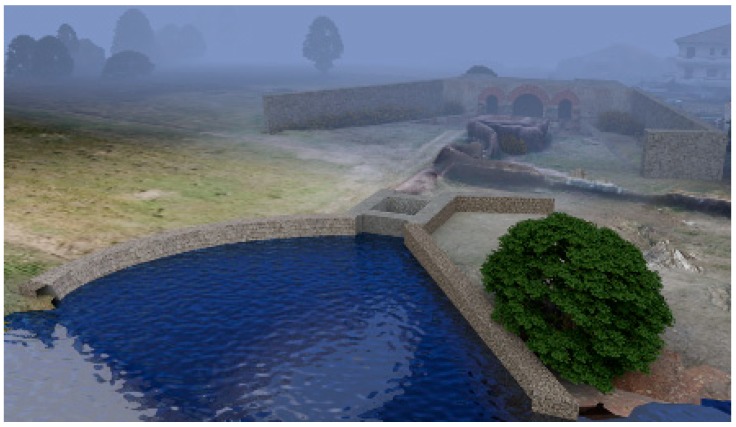
Rendering of *incile* as it would have appeared today if it would not have been destroyed.

Then the 3D mesh, with the real texture of the territory applied, was generated. On this mesh, the virtually reconstructed *incile* has been relocated, so that the ancient *incile* is inserted in the territory as it appears today. Its position error should be limited to a few meters. The final renderings of how the reconstructed *incile* could appear nowadays are reported in Figure 12. In the construction of this picture, the point of view of the representation of Piranesi (Figure 5) and the same level of the water have been used. 

As regards to materials and textures for the *incile*’s reconstruction, they have been chosen taking into account what can be deduced by the available iconographic references and in compliance with the typical building technologies used in Roman times for similar works. In particular, the texture applied to the 3D models comes from a reworking of pictures of the Cunicolo Maggiore (the monumental tunnel connected to the Roman emissary) and from the modern Torlonia *incile*, where the same type of materials can be found. The textures from the Cunicolo Maggiore represent the uncertain Roman stone masonry with appeals in rows of bricks partially used in the ancient *incile* (*opus mixtum*). The carved stones of the walls of the modern *incile* with indentations are a reference for the limestone material used for reconstructing the entrance of the tunnel in the ancient *incile*.

**Figure 13:**
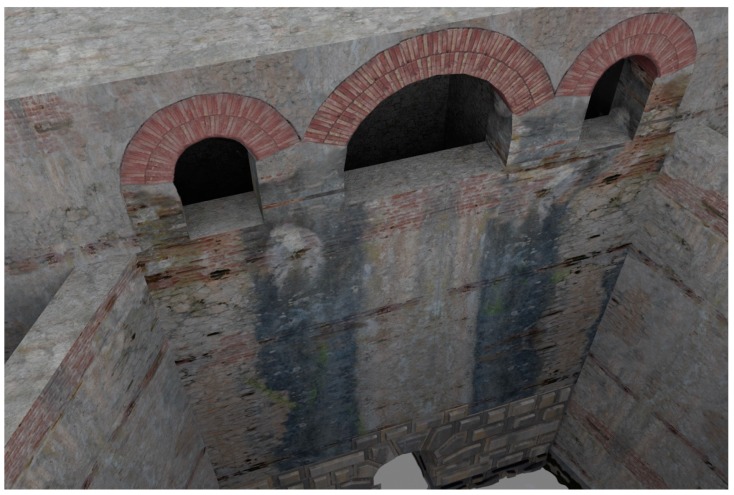
Detail of the virtual reconstruction of the trapezoidal tank.

**Figure 14:**
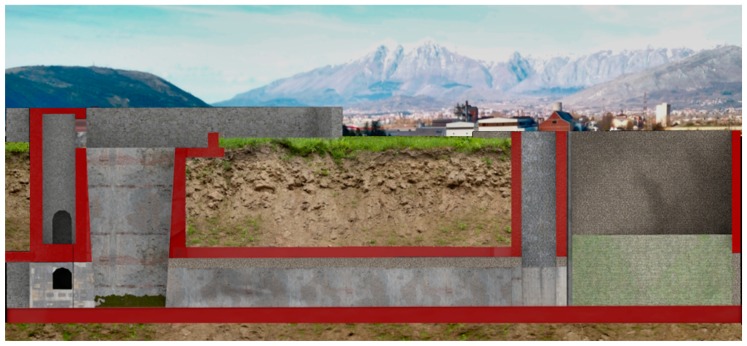
Longitudinal section of the Roman *incile* in a current setting performed by the 3D virtual reconstruction.

In some cases, the textures have been constructed by composing single elements; this is the case with the arches with two orders of bricks (Figure 13) or with the stone coverings made in *opus reticulatum*. Figure 14 shows the longitudinal section of the Roman *incile* in a current setting performed by the 3D virtual reconstruction.

All renderings have been generated by using Cinema 4d R.20 (developed by Maxon Computer GmbH, Max-Planck-Str. 20, 61381 Friedrichsdorf Germany).

## 5. Results and Conclusions

The virtual reality applied to CH is usually used for aesthetic reconstructions, 3D visualization, and documentation of architectural opera or landscape contexts, nowadays partially or totally destroyed. In some cases, when ruins or original ancient elements are found, a virtual anastlylosis can be performed. In such situations, only the remaining original elements are used for the reconstruction. Other elements, limited to those necessary to connect the original fragments among them, may be added; when that happens, these elements must be, anyway, evidenced and made recognizable as replacement material. In virtual anastylosis, in fact, the main inputs come from the existing ancient findings, which fundamentally drive the reconstruction process.

In this work, the reconstruction of an ancient opera, for which no original fragments or ancient ruins are available, is performed. Heterogeneous data coming from different sources were collected to provide a 3D virtual reconstruction of a hydraulic opera that is not visible anymore. The reconstruction of the ancient *incile* of Fucino Lake described here, results from a reverse engineering process that scientifically extracts knowledge and functional information of the opera from territory data, historical information, and archival sources documentation, such as the drawings of the Roman *incile* performed in the 19th century. The territory data, useful to correctly place the *incile*, were acquired with traditional techniques and with UAV photogrammetry. A specific experiment verified that the quality of the obtained point cloud is suitable for the aims of this paper. 

**Figure 15:**
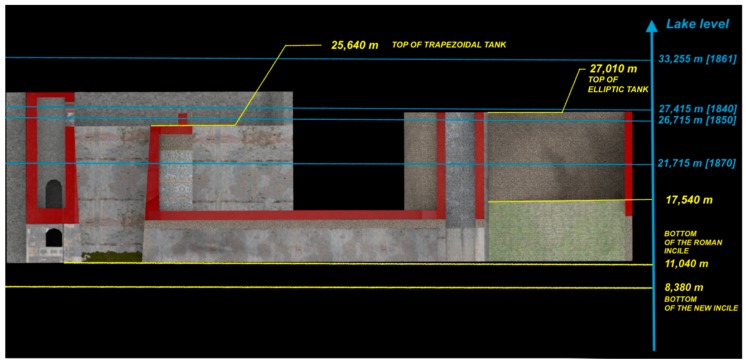
Longitudinal section of the Roman *incile* with the water levels of Fucino Lake in the 19th century.

This is a new approach that, by drawing important information from the functional analysis of the hydraulic opera, allows reconstruction of the ancient *incile*, while investigating its original functionalities and how it was conceived by the Romans. This cannot be achieved by a traditional 2D representation of the structure. Such is the case with the functional interpretation of two asymmetric lateral winzes shown in Figure 2. Based on 3D virtual reconstruction of the i*ncile,* these winzes could be interpreted as channels probably used in one of the preliminary phases of the drainage work, in order to conduct the waters to the underground emissary. Furthermore, important information on the *incile*’s functionalities can be obtained by examining some data relating to the water levels of Lake Fucino, especially during the drainage phase. Figure 15 shows the water levels measured in some specific situations on the *incile*’s longitudinal section of Figure 14. Such information, combined with the virtual representation of the *incile* and the territory are useful for analyzing the functionalities of the individual parts of the hydraulic architecture and speculating on how they worked, or why they were built. The lake drainage required many years, as demonstrated also by the 19th century works. At that period, the *incile*’s inlet operas changed a lot. The first drainage, performed in 1862, was executed by backlogging a gate to the emissary of about 400 m with respect to the ancient *incile* and using a canal placed many meters above the bottom of the Roman tanks. Again, in the 19th century and in at least another two phases of drainage works, the inlet also changed in its location to take into account the variations of the lake extension during drying. The same problems and the same needs certainly occurred during the ancient Roman works so that the ancient *incile* must be analyzed considering that it was a work in progress. Only in this way, is it possible to understand its complexity and the reason why at least three tanks were built over time. 

In conclusion, the peculiarity of the approach proposed here is that using the non-uniform data coming from different sources, not for a simple external reconstruction of the site but for investigating the original functional aspects, led to the site realization in the way documented by the various sources. In an era of data dissemination and sharing via web and social networks, it will becoming increasingly easier to find data on the territory, historical information, and archive documentation, so that it is foreseeable that studies aimed at the reconstruction of the sites, of which each trace has been lost, will be increasingly encouraged.

Future works, therefore, could use this 3D reconstruction of the *incile* for generating some simulation models aimed at investigating the different hypotheses that can be formulated on the functionalities of the various parts of the structure.

## Figures and Tables

**Table 1:** Technical specifications of the UAV used for the survey

**Type of UAV**	
*Micro UAV Quadricopter*	
**Optical sensor**	
**Parameter**	**Value**
Focal length	35 mm
Sensor dimensions	6.17 mm × 4.55 mm
Resolution	13 Mpixel
Pixel dimension	10 µm
FoV	78°

**Table 2:** Flight parameters.

Parameter	Value	Parameter	Value
Flight altitude from SP	70 m	GSD	4 cm
Front overlap	75 %	GCPs	4
Side overlap	65 %	Viewing angle θ	0 deg
Flight speed	6 m/s		

**Table 3:** Settings used in Pix4D software.

Point Cloud.	3D Mesh
Image scale = 1:1	Maximum octree depth = 14
Points density = Optimal	Texture size = 16,384 × 16,384
Minimum number of matches = 3	Decimation criteria = qualitative (sensitive)
Tie-points = 10,000 Coordinate accuracy = High	

**Table 4:** Altitude comparison between the CPs.

Point	Altitude Survey 1996	Measured Altitude	Error
1	666.9 m	666.9 m (RIF)	-
2	674.0 m	673.8 m	0.20 m
3	677.4 m	677.45 m	0.05 m
4	690.5 m	690.1 m	0.40 m
5	699.3 m	698.5 m	0.80 m
